# Extensive Abdominal Skin Necrosis Following Anterior Component Separation for a Large Ventral Hernia: A Case Report

**DOI:** 10.3389/fsurg.2021.779046

**Published:** 2021-12-17

**Authors:** Branko Bakula, Marko Sever, Andrija Karačić, Mirko Bakula, Martin Grbavac, Ivan Romic, Ante Bogut, Zvonko Zadro

**Affiliations:** ^1^Department of Surgery, University Hospital Sveti Duh, Zagreb, Croatia; ^2^Department of Urology, University Hospital Centre Zagreb, Zagreb, Croatia; ^3^Department of Surgery, University Hospital Centre Zagreb, Zagreb, Croatia; ^4^Department of Gastroenterology, University Hospital Mostar, Mostar, Bosnia and Herzegovina

**Keywords:** hernia recurrence, wound dehiscence, anterior component separation (ACS), postoperative complication, skin ischemia

## Abstract

**Introduction:** Hernia surgery is one of the most common operative procedures, performed in about 20 million cases per year all over the world, with ventral hernia accounting for about 30% of the cases. Although the introduction of the anterior component separation (ACS) method, popularized primarily by Oscar Ramirez, has greatly facilitated the closure of the largest abdominal wall defects, the 30-year experience in this technique has pointed to the risk of ischemic skin complications consequential to the major subcutaneous tissue dissection required. The aim of this case presentation of a patient who developed extensive necrosis of the abdominal wall skin following ACS procedure is to emphasize the importance of preserving rectus abdominis perforator blood vessels in order to preserve skin vitality.

**Case Presentation:** We present a case of a 58-year-old female patient with a large recurrent ventral hernia. The hernial defect was closed by placing a large (30 × 25 cm) polypropylene mesh in the retro-rectus space using the Rives-Stoppa technique. To facilitate upper fascia closure ACS according to Ramirez was performed bilaterally. The rectus perforator vessels were not preserved. Recovery of the patient was complicated with the extensive abdominal skin necrosis which was successfully treated with negative pressure wound therapy.

**Discussion:** Transection of the musculocutaneous perforators of the epigastric artery during ACS results with the compromised blood supply of the abdominal skin depending solely upon the intercostal arteries. Skin ischemia following ACS is a serious complication that can be presented with extensive necrosis associated with high morbidity and even mortality, while the treatment is long lasting, complex, and expensive. Considering the ever-increasing prevalence of large ventral hernias, ever greater popularity of the ACS technique, and the growing proportion of surgeons performing large ventral hernia operations independently, we think that the role of preserving perforated rectus vessels has not been emphasized enough. Therefore, the objective of this case study is to stimulate surgeons to preserve skin vascularity and promote it in their routine in order to avoid these severe postoperative complications.

## Introduction

Hernia surgery is one of the most common operative procedures, performed in about 20 million cases per year all over the world, with the ventral hernia accounting for about 30% of the cases (1). According to the European Hernia Society, ventral hernias are divided into primary hernias and incisional hernias (2). It is estimated that 10–15% of patients with laparotomy will develop hernias during their lifetime. This risk rises to 23% in the patients whose recovery was complicated by infection of the laparotomy wound. Thus, 89% of incisional hernias occur after laparotomy, 5% after laparoscopy, while 6% are associated with a stoma (3, 4).

The main risk factors for the occurrence of ventral hernia are obesity and age. Considering the pronounced population aging owing to the modernization of the society, the problem of obesity grows to the extent of an epidemic, while the rate of abdominal operative procedures increases with advances in diagnostics and availability of healthcare, clearly indicating that ventral hernias are becoming a greater public health issue.

Other clinical conditions that increase the risk of incisional hernia are chronic obstructive pulmonary disease (COPD), diabetes mellitus, anemia, using vasopressors and steroids, renal failure, malnutrition, and malignant disease (5).

In the past, simple hernial orifice suturing was the only method available for the management of ventral hernia, which was expectedly associated with a high rate of recurrence due to the high-suture tension. However, the management of ventral hernias has been revolutionized with the advent of synthetic mesh and the development of the component separation technique. Abdominal wall mesh reinforcement combined with the mobilization of the abdominal wall musculofascial flaps enables tension-free closure of major hernial defects, which has led too much more successful treatment of ventral hernias and a great reduction in the recurrence rate.

Although the introduction of the anterior component separation (ACS) method, popularized primarily by Oscar Ramirez, has greatly facilitated the closure of the largest abdominal wall defects, the 30-year experience in this technique has pointed to the risk of ischemic skin complications consequential to the major subcutaneous tissue dissection required (6, 7).

The aim of this case presentation of a patient who developed extensive necrosis of the abdominal wall skin following procedure of ACS is to emphasize the importance of preserving the rectus abdominis perforator blood vessels, as well as the importance of timely initiation of the adequate modality of treatment of the postoperative ischemic skin complication.

## Case Presentation

We present a case of a 58-year-old female patient with a large recurrent ventral hernia. Six years before, the patient had been operated on for the umbilical hernia, with the simple repair without a mesh. The patient was an active smoker who suffered from morbid obesity with a body mass index of 43 kg/m^2^ and COPD as comorbidities relevant for this case report.

The patient was introduced to the surgeon during hospitalization at the gastroenterology department where a diagnostic workup due to a clinical picture of chronic small bowel obstruction was conducted. While taking the anamnesis, the patient reported frequent abdominal cramps, swelling, and pain in the area of the hernia that had intensified in the last few weeks. The physical examination revealed a large irreducible ventral hernia in the lower abdomen that was quite painful on palpation, but soft and, at that time, without signs of incarceration or strangulation. Taking into account the clinical picture with threatening hernia incarceration, the surgeon did not opt for preoperative optimization of the patient in terms of smoking cessation and starting a weight loss program but made an indication for semielective surgery.

On operative procedure, greater omentum, part of the transverse colon, and a cluster of small bowel loops with signs of chronic obstruction were found as hernial content. After adhesiolysis hernial content was reduced into the abdominal cavity. Hernial defect measuring about 7 cm in diameter and about 15 cm in the vertical line with significant rectus diastase in the supraumbilical part of the abdomen was revealed. Using the Rives-Stoppa technique a wide retromuscular space was created. Lateral dissection boundaries of this space were perforating neurovascular bundles in the area of the lateral edges of the rectus muscle on both sides. The posterior fascia was easily closed using also a portion of the hernia sac to bridge the defect between the posterior rectus sheaths. A 30 × 25 cm polypropylene mesh was placed in the retromuscular space ensuring adequate mesh overlap over the edges of the hernia defect of a minimum of 5 cm in all directions.

When we observed that the anterior fascia, due to the size of the defect and decreased abdominal wall elasticity, would not close entirely and cover the mesh, we opted for rectus mobilization by the ACS method to avoid bridging. Upon extensive dissection of the anterior abdominal wall subcutaneous space without preservation of the rectus perforator vessels, relaxing incisions of the external oblique muscle aponeurosis were performed. Using the Ramirez technique, long longitudinal incisions of aponeurosis were made bilaterally, adjacently to the semilunar line, extending from the costal arch to the groin. This procedure resulted in the considerable mobilization of the vital musculofascial flap medially, and the hernial defect was closed at the midline without tension. Then, four redon drains were placed, i.e., 2 in the retrorectus space and another 2 in the subcutaneous space.

**Figure 1:**
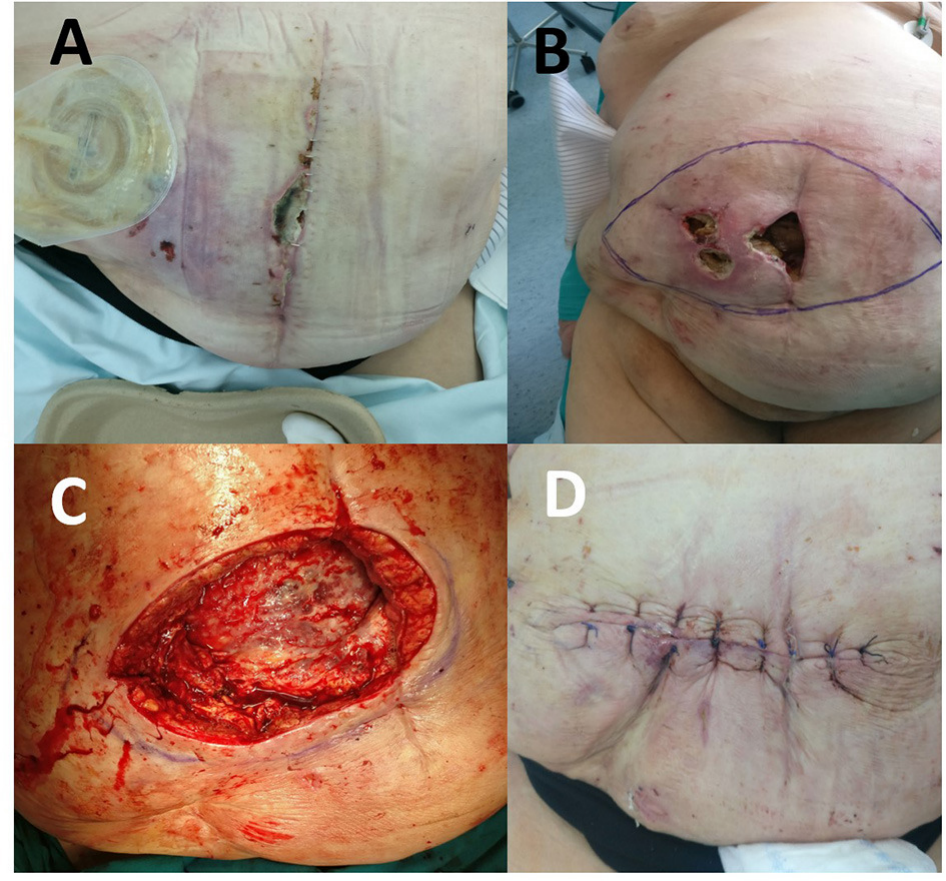
**(A)** Ischemic lesion on the 8th postoperative day. **(B)** Two full-thickness skin necrotic foci paramedially on the 22nd postoperative day. **(C)** View of the abdominal wall after necrectomy on the 22nd postoperative day. **(D)** View of the abdominal wall after the wound was primarily closed.

The postoperative course was complicated by skin ischemia. Ischemic lesions of the abdominal wall skin on the right with signs of necrosis along the midline were observed already on day 8 (Figure 1A). On postoperative day 11, multi-slice computed tomography (MSCT) of the abdomen was performed because of the ever more abundant wound discharge. MSCT findings showed a large subcutaneous seroma, a normal musculofascial component of the abdominal wall, appropriate mesh position, and normal intra-abdominal status. Percutaneous puncture of seroma was performed and about 800 ml of clear seroma was evacuated. During the next 10 days, ischemia progressed, along with the development of another two full-thickness skin necrotic foci paramedially (Figure 1B). Considering the relatively strict demarcation area of necrosis, we opted for the operative procedure of necrosectomy.

**Figure 2:**
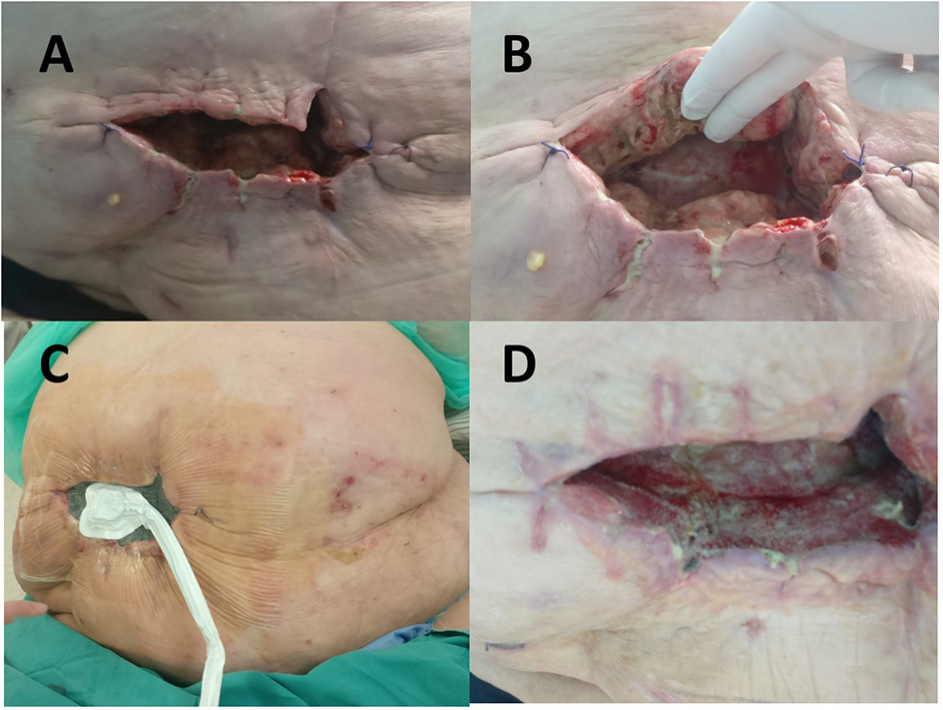
**(A)** Wound dehiscence on the 4th postoperative day after necrectomy with primary closure. **(B)** Fibrin deposits with zones of the necrotic tissue in the wound. **(C)** Negative pressure wound therapy delivered in continuous mode with negative pressure maintained at −100 mm Hg was initiated on the 33rd postoperative day. **(D)** View of the wound 2 weeks after negative pressure wound therapy (NPWT) administration with signs of considerable improvement seen in cavity reduction, decreased wound discharge, and formation of healthy granulation tissue.

Following abdominal wall necrosectomy with a safety margin of healthy tissue and considering an appropriate amount of vital residual abdominal skin, as well as the absence of signs of local tissue infection or mesh infection, primary wound closure was performed in consultation with a plastic surgeon (Figures 1C,D). As early as day 4 of the second operation, increased wound discharge and signs of skin wound dehiscence occurred, which required removal of skin sutures (Figures 2A,B). Then, a wound dressing with a hypertonic solution was applied for a week.

**Figure 3:**
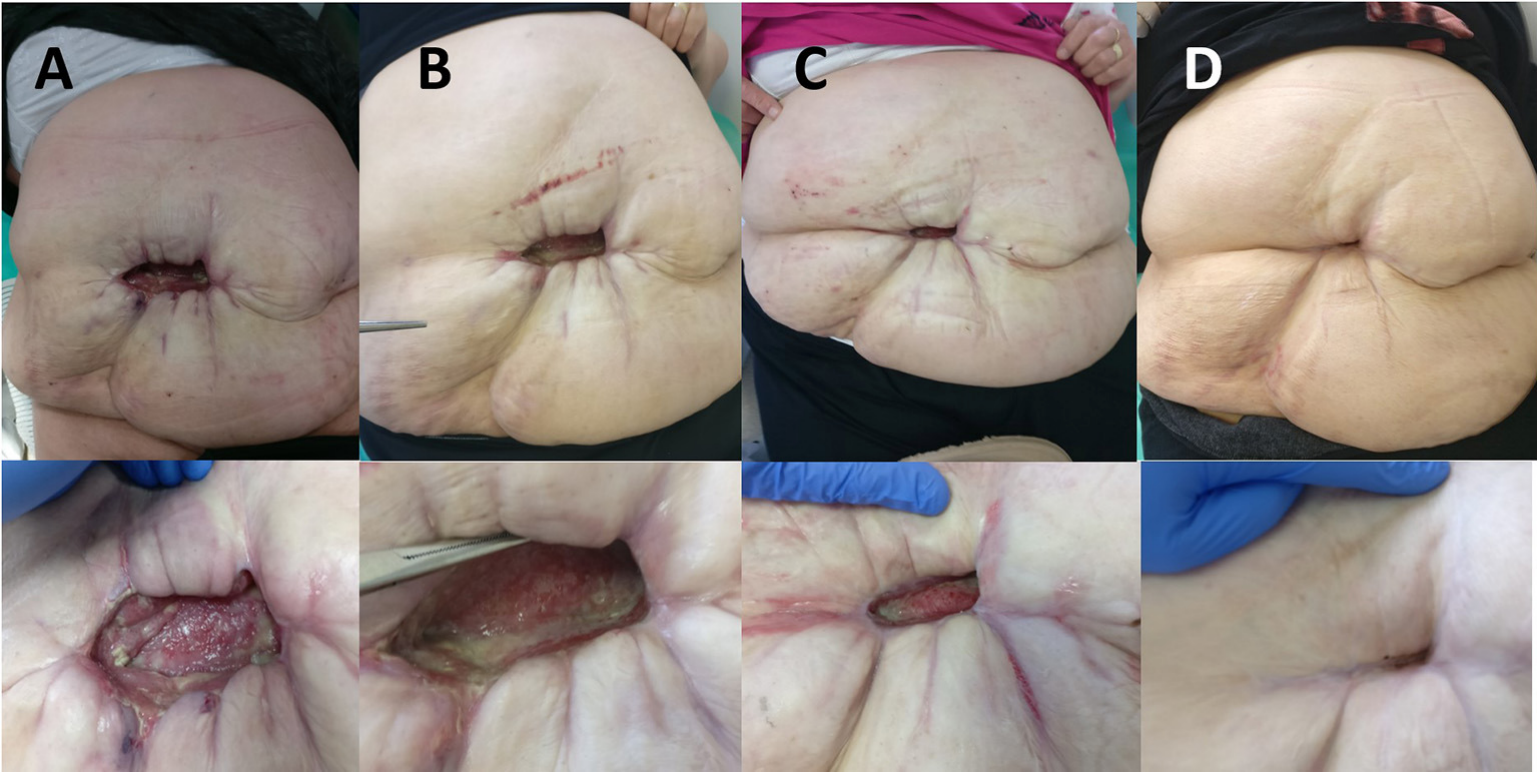
**(A)** View of the abdomen 3 months after the initial surgery. **(B)** View of the abdomen 4 months after the initial surgery. **(C)** View of the abdomen 6 months after the initial surgery. **(D)** View of the abdomen 10 months after the initial surgery.

When inflammation subsided, negative pressure wound therapy (NPWT) with the “Renasis Ez Max VAC® system” (Smith & Nephew, Mississauga, Canada) was initiated (Figure 2C). NPWT was delivered in continuous mode with negative pressure maintained at −100 mm Hg. Dressing in the form of a sponge of polyurethane black hydrophobic foam was changed every third day. After 2 weeks of NPWT administration, considerable improvement was recorded in wound cleaning and formation of healthy granulation tissue (Figure 2D). NPWT was continued for the next 2 months, which resulted in further improvement of condition of the patient, along with decreased wound discharge and cavity reduction. The wound swab obtained twice during dressing change was sterile. The patient was discharged from the hospital and regular changing of silver-impregnated antimicrobial wound dressing (Aquacel Ag, ConvaTec, Reading, United Kingdom) was continued in ambulatory care that led to complete wound closure in 7 months (Figures 3A–D).

## Discussion

In the past, the management of abdominal hernia was rather frustrating challenge for surgeons, associated with a high-recurrence rate. As early as the end of the nineteenth century, Theodor Billroth anticipated that the issue of hernia treatment could not be managed without the use of a specific synthetic material (8). Since that time, attempts have been made with various materials such as metal, cotton, silk, etc., however, without success due to the high rate of complications, foreign material rejection, inadequate firmness, and infections. More than half a century had to elapse before the discovery of an ideal material for a mesh that could be used in hernia surgery. The ideal mesh should have the following characteristics: infection resistance, inertness, maintaining long-term firmness, flexibility to avoid breaks, the capacity of fast tissue ingrowth, and absence of carcinogenicity.

In 1958, Francis Usher proposed the use of polypropylene mesh that had almost all of the favorable characteristics listed earlier, thus having introduced hernia surgery into a new, more successful era (9). Polypropylene mesh with its pores stimulates the formation and ingrowth of the fibrous collagen tissue around the mesh, resulting in the formation of a firm sheet in the abdominal wall.

In the 1960s, Jean Rives and Rene Stoppa, French surgeons, laid the groundwork for the modern management of ventral hernias. In their independent researches, they proposed sublay mesh placement as the ideal one. The idea originated from their own great experience of placing a large mesh in the preperitoneal space of the lower abdomen for treatment of groin hernias (10, 11). Later, they proposed mesh placement in the retro-rectus area in the management of all ventral hernias located above the arcuate line, thus obviating technically demanding preparation of the thin and fragile preperitoneal space between the peritoneum and posterior rectus sheath (12).

With some minor variations, the currently standardized Rives-Stoppa technique is based on several crucial steps, as follows:

- hernia dissection and repositioning to the abdominal cavity;
- creating a broad retro-rectus area (and/or preperitoneal space below the arcuate line) for mesh placement, posteriorly covering the primary hernial defect, always extending over the hernial defect margin by at least 3 cm; and
- closure of the hernial orifice by covering the mesh with the anterior fascia and the rectus muscle (13).

Although other mesh placement locations have been maintained to date (onlay, inlay, and underlay), many years of experience have shown that the sublay space is indeed mechanically the most advantageous. One of the main reasons for this is certainly the fact that in this way the ingrowth of fibrous tissue into the mesh from two firm tissues that are primarily intended for load-bearing—the posterior sheath of the rectum and the rectus muscle itself, is ensured. Among the numerous studies that confirm this theory, a large meta-analysis from 2016 conducted by Holihan (“Mesh Location in Open Ventral Hernia Repair: A Systematic Review and Network Meta-analysis”) stands out (14). This case study included an analysis of 41 scientific papers with more than 5,200 patients operated on for ventral hernia with mesh placement. A much lower rate of hernia recurrence and surgical site infection was found with the sublay method compared with the onlay, inlay, and underlay method (7 vs. 16.5, 30.2, 14.7%, and 3.7 vs. 16.9, 31.3, and 16.7%, respectively).

In the case of medium large and large ventral hernias, closure of the superior fascia by the Rives-Stoppa technique may be more challenging as compared with the closure of the mobilized posterior fascia because this technique does not enhance superior fascia mobility. The more so, with incision of the upper flap of the rectus sheath, which is often performed more laterally from the medial edge, the material to be used in the superior fascia closure is lost. Therefore, the superior fascia is frequently closed under some tension or cannot be closed completely, leaving a small segment unclosed as “bridging.” There is no strict consensus on the acceptable maximal bridging width; however, experience has shown that in the case of appropriate large mesh sublay placement, bridging of up to 4–5 cm can be tolerated with satisfactory long-term results.

In this paper entitled “*The treatment of complicated groin and incisional hernias*” from 1989, Stoppa emphasized the importance of upper fascia closure over the retro-muscularly placed mesh, even if requiring relaxing incision of the superior rectus sheath (15). Although he did not describe the technique in detail, he anticipated the ACS technique published a year later by Oscar Ramirez, an American plastic surgeon.

In 1990, Ramirez described a technique of musculofascial flap mobilization by relaxing incisions of the external oblique muscle aponeurosis, thus having greatly upgraded ventral hernia surgery (6). Although the idea presented in his original paper entitled “*Components separation method for closure of abdominal-wall defects: an anatomic and clinical study*” was based on tension-free closure of the major hernial defects without using a mesh, currently, this technique is mostly employed as a necessary and very useful step in major ventral hernia repair by the methods that include sublay or underlay mesh placement.

The ACS technique is based on a long incision of the external oblique muscle aponeurosis along the rectus muscle lateral edge (from the costal arch to the inguinal area). This procedure results in extensive medialization of the vital flap of the rectus muscle with its sheath medially, even up to 10 cm at midline, thus enabling closure of very large hernial orifices that had previously presented unsolvable obstacles to surgeons.

Summarizing the current guidelines for the treatment of ventral hernias, five basic rules can be identified that the surgeon should follow in order to reduce the recurrence rate and avoid postoperative complications:

1. Preoperative patient optimization (e.g., weight loss, smoking cessation, regulation of glycemia, improving nutritional status, and improving oxygenation in patients with chronic hypoxia).
2. Centralization and reapproximation of the rectus muscles under physiologic tension when feasible using component separation technique when appropriate.
3. Use of appropriate reinforcement material (permanent synthetic mesh for non-contaminated situations) for all defects larger than 2 cm.
4. The recommended space for placing the mesh is “sublay” (retromuscular/preperitoneal) (using the Rives-Stoppa technique or transversus abdominis release technique).
5. In contaminated fields or in patients with increased risk for surgical site infection, the use of biological mesh or primary non-mesh closure with planned delayed definitive reconstruction is recommended (16–18).

Despite the fascinating results in terms of rectus mobilization achieved by the Ramirez method, it should be noted that initially, the procedure was burdened by complications such as ischemia or skin necrosis due to the need of extensive subcutaneous tissue undermining to reach lateral edge of the rectus muscle (19). Namely, in his original technique description, Ramirez failed to consider preserving skin vascularity *via* rectus abdominis perforator vessels. It may not pose a problem in young and generally healthy individuals but the risk of ischemic skin complications following ligation of the skin supplying arteries increases substantially in patients with impaired skin vascularity such as obese patients, patients with COPD, diabetic patients, and those with abdominal wall scars from previous operations (20).

That is why the American surgeon Saulis deserves to be mentioned here because he recognized the importance of preserving rectus abdominis perforator arteries and their predominant location at the level of the umbilicus. In 2002, in his paper called *Periumbilical rectus abdominis perforator preservation significantly reduces superficial wound complications in separation of parts hernia repairs* he described his modification of Ramirez technique, where periumbilical rectus abdominis perforator arteries responsible for skin supply are preserved during tissue dissection (21). The article provides a thorough description of the technique where complete subcutaneous tissue is preserved in 5–6 cm around the umbilicus, whereas incision of the external oblique muscle aponeurosis is performed through a tunnel of subcutaneous tissue connecting two dissection areas below and above the umbilicus. In this case study, Saulis compared the results obtained in two groups of patients operated on by classic Ramirez technique and modified technique preserving rectus abdominis vessels. He found a significant difference in the rate of postoperative ischemic skin complications, whereas there was no difference in the rate of recurrence and other postoperative complications. Skin ischemia or necrosis occurred in as many as 20% and only 2% of patients operated on without and with rectus abdominis perforator vessels preservation, respectively.

Skin ischemia following ACS is a serious complication that is presented with extensive necrosis associated with high morbidity and even mortality, while the treatment is long lasting, complex, and expensive.

Analyzing the presentation of our case, any surgeon with experience in the treatment of ventral hernias would easily notice certain cardinal mistakes that occurred during the decision for the type of surgery and also during the management of postoperative complications. These mistakes resulted in the development of extensive abdominal skin necrosis, but also an unnecessary prolongation of treatment of the resulting complication.

That is why the aim of this case study is to draw attention to the following mistakes in treatment that can easily happen to a younger surgeon or a surgeon who has no experience with the treatment of complex ventral hernias:

1. Considering that the patient had three risk factors that significantly compromise the blood supply to the skin of the abdominal wall (COPB, smoking, and obesity), we believe that the ACS technique which significantly increases the risk of ischemic complications should have been avoided. Namely, despite the impossibility of complete coverage of the mesh with upper fascia, it should have been assessed intraoperatively that the risk of ischemic complications after ACS is higher than the risk of complications due to residual “bridging” of 4–5 cm in one part (e.g., mesh infection, seroma formation, and hernial recurrence).
2. Once it has been already decided to perform ACS, it is unquestionable that one of the techniques which involve preserving the rectus abdominis perforator arteries should have been used. In addition to the Saulis method of preserving skin vascularity, some other techniques have also been described to dates, such as relaxing incisions through separate skin incisions in the projection of the lateral rectus edge and endoscopic incision of the external oblique muscle aponeurosis (22).
3. In the postoperative course, primary wound closure after necrectomy was a mistake that delayed recovery, delayed the onset of NPWT, and increased the risk of developing infectious complications. Such a wound, with a still-present inflammatory reaction and increased secretion, had no chance of healing primarily closed, and dehiscence was inevitable.
4. Given the extent of the skin defect and the presumed need for long-term therapy, outpatient NPWT treatment should have been organized to reduce treatment costs, hospitalization days, but also to reduce the risk of developing nosocomial infections.
5. During the postoperative treatment of skin necrosis, the application of instillation NPWT (NPWTi) should have been considered. NPWT has proven to be one of the most successful modalities of treatment for complicated wounds. By increasing local blood flow, reducing bacterial colonization, and inducing granulation and angiogenesis, NPWT strongly facilitates wound contraction and cavity reduction (23). The modification of classical NPWT is instillation NPWT in which during intermittent NPWT, in the phase of pausing negative pressure, a topical antimicrobial wound solution is instilled into the wound which stimulates the formation of granulations. The solution soaks the complete wound, and thus, penetrates into the deepest recesses of the wound where it facilitates mechanical debridement of necrotic tissue which is removed from the wound during the negative pressure phase. Given the lengthy process of demarcating skin necrosis and the existence of deep undermined pockets in the wound in our patients, we believe that NPWTi would significantly accelerate wound healing (24).

Some of the approaching alternatives to traditional wound-healing treatments such as the use of bioengineered skin substitutes should be mentioned here. This skin substitutes greatly help with the closure of a large skin surface defects. In the case of full-thickness skin defects, as is the case with our patient, an acellular dermal regeneration template (aDRT) would be used to fill the skin defect after necrectomy. aDRT is a porous, resorbable natural or synthetic polymeric biomaterial with a 3D structure, and its basic task is to take on the role of a temporary dermis into which the connective tissue of the recipient would grow in order to form a neodermis (25). In the case of our patient, the application of this type of treatment would be considered at a later stage of the wound-healing process when, with the help of NPWT, the volume of the cavity and wound secretion were already significantly reduced and fresh granulations were produced. Then, the use of DRT with the creation of adequate neodermis and the final covering of the wound with a split-thickness skin graft would potentially further shorten the treatment of our patient.

This case study clearly exemplifies the role of preserving skin vascularity on subcutaneous space dissection using the ACS technique. Our own experience before this case study presented refers to more than 15 operations according to Ramirez with the rectus perforator vessels sacrificed, none of these were associated with ischemic complications. It is just the latter results that might prove quite tricky for a young surgeon, encouraging him/her to continue using this technique after several successful operations without preserving skin vascularity. The patient presented here had strong predisposing factors to develop this complication (COPD, obesity, and smoking), whereas the development of such ischemic skin complication is much less likely in young and healthy individuals. Despite that, we do believe that the procedure of preserving rectus abdominis perforator vessels on performing ACS technique should be implemented as a routine and unavoidable part of this highly useful method not only for high-risk patients but also for all patients.

**Figure 4:**
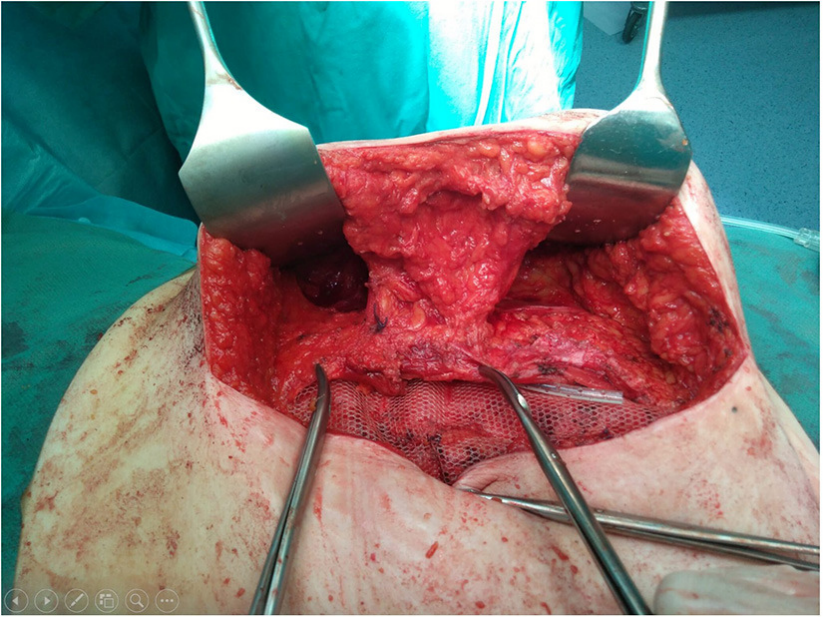
View of the operative field after anterior component separation (ACS) with periumbilical rectus abdominis perforator vessels preservation according to Saulis.

In our institution, following the complication described, we have accepted the method of Saulis as a standard procedure on performing ACS technique, with no ischemic skin complications recorded in more than 40 patients operated on so far (Figure 4). This mode of preserving rectus perforators requires patience and caution, while only slightly prolonging the procedure, but it is quite easy to master.

## Conclusion

Transection of the musculocutaneous perforators of the epigastric artery during ACS results with the compromised blood supply of the abdominal skin depending solely on the intercostal arteries and sometimes it is just not enough.

Considering the ever-increasing prevalence of large ventral hernias, the ever-greater popularity of the ACS technique, and the growing proportion of surgeons performing the large ventral hernia operations independently, we think that the role of preserving perforated rectus vessels has not been emphasized enough. Therefore, the objective of this report is to stimulate surgeons to preserve skin vascularity and promote it in their routine in order to avoid these severe postoperative complications.

We also want to emphasize that for long-term good results in the treatment of complex ventral hernias, a comprehensive approach to treatment that includes preoperative optimization of the patient, careful setting of indications for a particular surgical technique, adequate surgical technique, and education on modern care of the postoperative ischemic or infectious skin complications is crucial.

## Data Availability Statement

The original contributions presented in the study are included in the article/supplementary material, further inquiries can be directed to the corresponding author/s.

## Ethics Statement

Written informed consent was obtained from the individual(s) for the publication of any potentially identifiable images or data included in this article.

## Author Contributions

BB initiated and performed the surgery. All authors were involved in the writing, reviewing, and editing of the manuscript.

## Conflict of Interest

The authors declare that the research was conducted in the absence of any commercial or financial relationships that could be construed as a potential conflict of interest.

## Publisher's Note

All claims expressed in this article are solely those of the authors and do not necessarily represent those of their affiliated organizations, or those of the publisher, the editors and the reviewers. Any product that may be evaluated in this article, or claim that may be made by its manufacturer, is not guaranteed or endorsed by the publisher.
